# Improving hypertension awareness and management in Vietnam through a community-based model

**DOI:** 10.1038/s41598-022-22546-w

**Published:** 2022-11-18

**Authors:** Helen McGuire, Truong Bui Van, Hien Le Thi Thu, Huyen Nguyen Thanh, Marge Murray, Jason Shellaby, Ann Aerts, Roshini George, Mary Hodges

**Affiliations:** 1 PATH, 455 Massachusetts Ave NW, Suite 1000, Washington, DC 20001 USA; 2 PATH, Ho Chi Minh City, Vietnam; 3 PATH, Hanoi, Vietnam; 4 PATH, Seattle, WA USA; 5 Novartis Foundation, Basel, Switzerland

**Keywords:** Public health, Population screening, Patient education, Hypertension

## Abstract

Hypertension prevalence in Vietnam is high, but few people are aware of their disease status. Detection, diagnosis, and treatment are limited, so new approaches are needed to improve awareness and manage the condition, especially at the primary health care level. The Communities for Healthy Hearts programme operated in four districts of Ho Chi Minh City, with the aim of increasing awareness and ensuring linkage to care. Interventions focused on extending services beyond health facilities to convenient community locations; training cadres of community volunteers to screen, refer, and follow up with clients; training health workers in facilities to diagnose, educate, and manage patients referred from the community; and supporting the process with digital case tracking. Community-based blood pressure screenings took place in client homes, commune health stations, and non-traditional sites such as coffee shops, dentists’ offices, marketplaces, neighbourhood watch/security posts, and tailor shops. In total, 121,273 adults aged 40 and older were screened. Of these, 25.1% had elevated blood pressure or a previous hypertension diagnosis and were referred to health facilities. Of those referred, 56.2% were confirmed to have hypertension, and of those confirmed, 75.2% were treated. Of those treated, 51.0% achieved blood pressure control, a favourable outcome over past programmes.

## Introduction

The World Health Organization (WHO) estimates that 17.9 million people die from cardiovascular diseases (CVDs) annually, and that CVD-related deaths will increase to 22.2 million annually by 2030 unless adequate preventive interventions and health care services are put in place^1,2^. Hypertension, or clinically high blood pressure (BP), is the prime risk factor for CVDs, mainly strokes and heart attacks, which account for 31% of total deaths globally^1^. WHO put forward ‘best buys’, such as integrating services into primary health care (PHC) as part of a universal health care package and ensuring that medicines and counselling are available to those at risk for CVDs^3^. WHO defines a best buy as a highly cost-effective intervention that is feasible and appropriate to implement within the constraints of local health systems^4^.

Although hypertension awareness and treatment have improved in high-income countries over the last decades, control remains a challenge. Low- and middle-income countries, meanwhile, are seeing growing incidence of the disease yet low rates of awareness and access to treatment^5^.

In Vietnam, hypertension is a disease of increasing public health concern. WHO’s noncommunicable disease (NCD) country profile shows that the prevalence of hypertension in Vietnam has been rising since 2000^6^. A recent systematic review identified a pooled prevalence of measured hypertension of 21.1% based on ten studies, and 18.4% based on three national surveys^7^. Two other studies confirmed high rates amongst people 40 years of age and older. The first study, which investigated a population aged 40–69 years in central Vietnam, showed the hypertension prevalence was 44.8%^8^. Another study, of people from 45 to 64 years old in Dien Bien Province, reported a prevalence of 35.5%^9^. Hypertension was found to be significantly higher in urban than in rural areas (32.7% and 17.3%, respectively) and increased with age in both men and women^10,11^.

In addition to the high prevalence of hypertension, the proportion of people in Vietnam aware of their disease status is unacceptably low. Also, the detection, diagnosis, treatment, and successful management of hypertension are limited. A 2012 study found that less than half of hypertension patients in the country were aware of their condition, and only 10.7% had achieved targeted BP control, defined as systolic BP below 140 mmHg and diastolic BP below 90 mmHg^11^. In 2015, the Ministry of Health conducted a national survey on risk factors for NCDs amongst adults aged 18 to 69 using the WHO STEPwise approach to Surveillance (STEPS) protocol. Data from the survey showed that, overall, 18.9% of a sample of 673 people had hypertension, but only 43.1% detected at screening were aware of their status. Of these, just 13.6% reported that their BP was being managed at a health facility^12^. These significant gaps in the hypertension treatment cascade are attributed to limited awareness amongst the population of how to prevent and control hypertension, substandard health care worker capacity to detect and manage hypertension, and lack of convenient access to routine BP screening and hypertension prevention and medication adherence counselling^8^.

Vietnam’s national strategy on prevention and control of NCDs (2015 to 2025) aims to detect 50% of those with hypertension and to treat and manage at least 50% those diagnosed^13^. The Ministry of Health also supports a national programme to improve hypertension prevention and control and has developed national standards and guidelines on management, reflecting an important commitment to address the disease and a critical foundation for action. The commune health station (CHS) plays an essential role in the national strategy to reduce the burden of hypertension by offering increased screening and early detection and the provision of a continuum of care for hypertension support and management^13^. In practice, however, Vietnam’s four-level health system is under-resourced to meet the needs of the population for hypertension prevention and care and has yet to demonstrate cost-effective service delivery models, particularly at the local level^14^.

Considering the increasingly enforced requirement that users of the nationally sponsored social health insurance plan initiate care at the commune or district level^15^, a critical opportunity exists to reinvigorate the commune health infrastructure. This will require CHSs to have well-trained personnel, be equipped with appropriate technologies and medicines, and be able to coordinate care with higher-level authorities and community case managers. This presents both an opportunity and an immediate challenge for patient-centred management of hypertension in Vietnam.

The Ministry of Health acknowledges that models are needed to demonstrate an effective and scalable hypertension detection, diagnosis, treatment, and management cascade. This involves increasing population awareness of CVD prevention as well as access to hypertension detection services; developing longitudinal care systems, including case management and patient registries; increasing health care worker capacity; and providing tools and guidance for those with hypertension to modify their behaviours and adhere to their treatment^16^. An analysis of the 2015 STEPS survey recommended that strengthening NCD services at the PHC level was urgently needed for early detection, diagnosis, treatment, and management of hypertension^12^. Against this backdrop, it should be noted that during the past decade, the private health care industry has grown exponentially, with a proliferation of private hospitals and clinics, small independent practices, and many businesses dedicated to health and wellness.

In 2016, PATH and the Novartis Foundation, in collaboration with Ho Chi Minh City Municipal Health Department and the Vietnam federal General Department of Preventive Medicine within the Ministry of Health, launched the Communities for Healthy Hearts (CH2) programme in Vietnam, a people-centred, community-based care model to introduce and test innovative approaches for BP control in the largest urban area of the country, Ho Chi Minh City. CH2’s unique multi-pronged model focuses on public-sector health services whilst also tapping into Vietnam’s growing private health care ecosystem to further extend the reach of PHC services. It uses digital technologies to ensure linkage to care and uses community collaborators to support awareness and to increase screening and retention in care. The impact of this model is seen in increased screening and retention rates as compared to national averages. For example, from June 2016 until the end of the project in 2019, 121,273 individuals representing 58% of adults aged 40 years and older in the project catchment area had received an initial hypertension screening^17^ as compared to 10%, as reported in the 2015 STEPS survey^12^.

Similarly, CH2 study results, as described in the results and discussion sections of this paper, indicated that a greater percentage of those diagnosed with hypertension initiated treatment and achieved BP control than was found in previous studies.

The paper explores the innovative components of the CH2 programme model and evaluates its operational aspects and effectiveness in expanding services for hypertension beyond the brick-and-mortar health care system.

## Materials and methods

### Programme model

**Figure 1:**
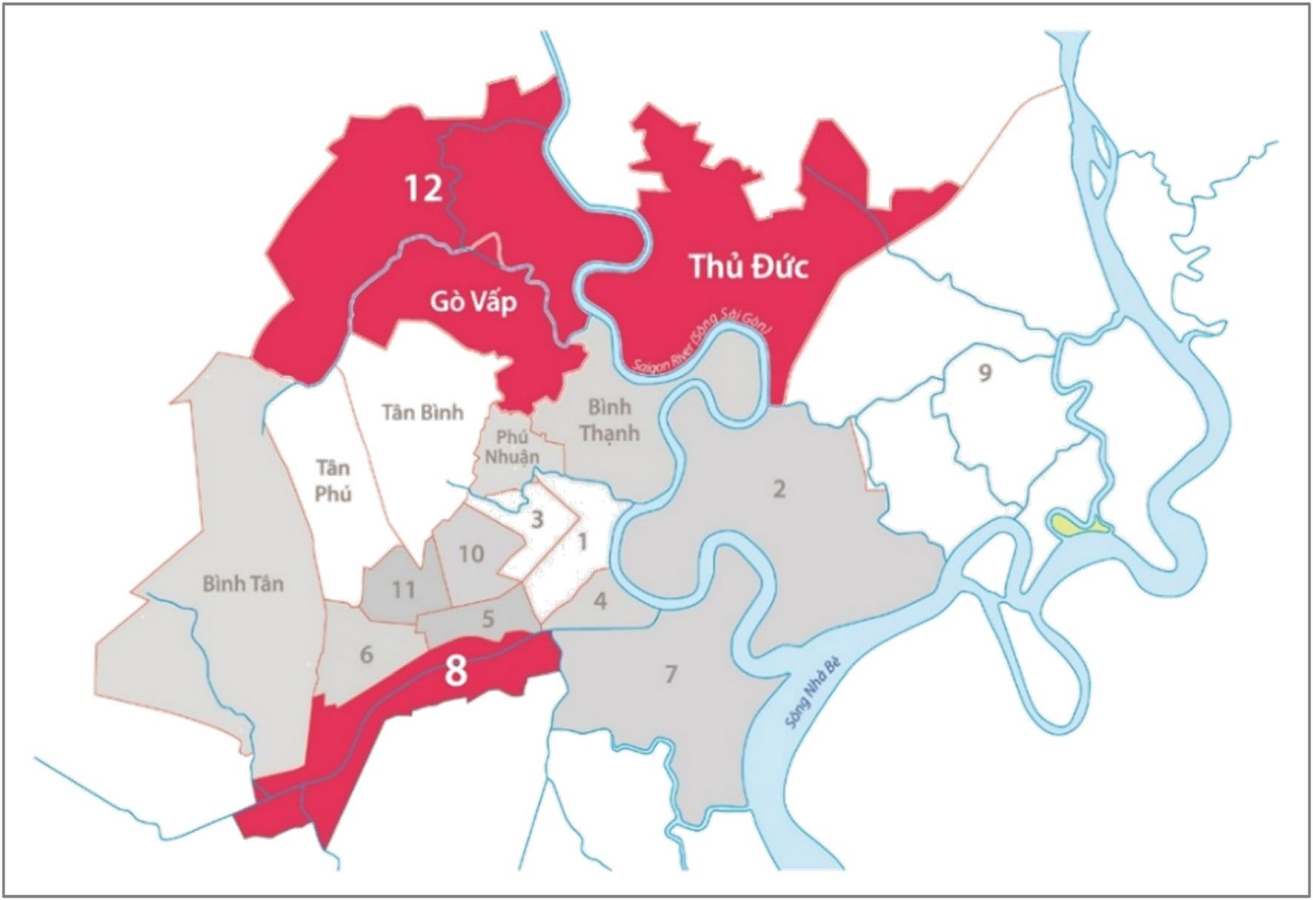
Ho Chi Minh City map with Communities for Healthy Hearts programme areas highlighted. *Source*: Adapted from Google images by Jayden Nguyen using Adobe Illustrator Version CC 2017.1 (21.1.0) to highlight the intervention districts. This image was adapted according to Google’s fair use principles.

CH2 was a three-year programme implemented from 2016 to 2019 through a partnership between PATH, national and local government leadership, and the Novartis Foundation. The programme tested a hypertension detection, diagnosis, treatment, and management cascade model in four districts in Ho Chi Minh City: District 8, District 12, Go Vap District, and Thu Duc District (Fig. 1). Ho Chi Minh City is a dynamic business and financial hub in the south of Vietnam with a population of nearly 9 million people. The selected districts, with a population exceeding half a million people, had the following characteristics that met the inclusion criteria that would support meeting project objectives: semi-urban district with low socioeconomic status that also had a large population of adults above 40 years old, commitment from district health leadership to address hypertension, and availability of a primary health care system as well as community network to support screening, referral, and linkage to care. According to 2019 census data, approximately 209,100 adults aged 40 and over reside in the catchment area^17^.

RCH2’s overarching goal was to improve hypertension awareness, treatment, and control amongst adults over 40. The project’s methodology centred around the objectives of increasing (1) the availability of patient-centred services for hypertension, (2) hypertension awareness, and (3) retention in care.

**Figure 2:**
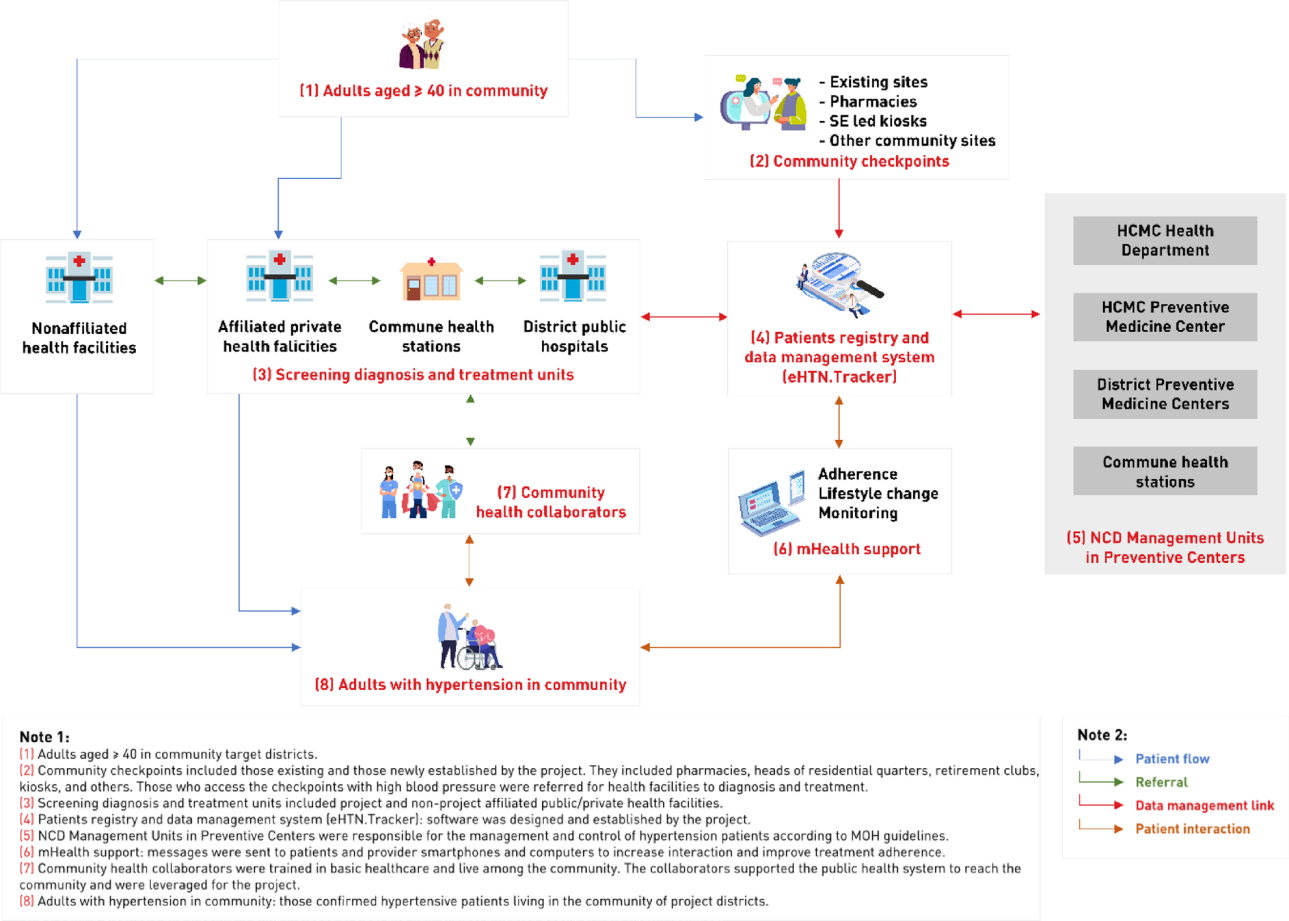
Diagram of the Communities for Healthy Hearts health care delivery model. *Source*: PATH.

The CH2 model^18^ (Fig. 2) was designed with Ho Chi Minh City health authorities and was based on findings from a targeted audience assessment^19^. It was informed by the global HEARTS technical package^2^, the Expanded Chronic Care Model^20^, and the Framework on Integrated People-Centred Health Services^21^. All experimental protocols performed in this study were in accordance with the ethical approval of Vietnam’s Institutional Review Board of the Center for Creative Initiatives in Health and Population (15 March 2018) and PATH Global Research Determination Committee. All methods were carried out in accordance with relevant guidelines and regulations. All participants provided informed consent.

CH2’s strategy had three main components that worked together to drive healthy behaviour change and uptake of hypertension services: establishing hypertension screening and referral services in the community; strengthening the capacity of community health volunteers and PHC workers to promote awareness of CVD risk and the importance of hypertension screening, treatment adherence, and healthy living; and directly implementing communication interventions with the target population.

To establish hypertension services in the community, close to where people live and work, CH2 engaged non-traditional health players from the community such as local shopkeepers to help screen and refer people with high BP to PHC services for hypertension diagnosis and treatment. The volunteers had no formal training in health care prior to CH2. One group of volunteers, known as ‘collaborators’, screened clients in their homes or at public places such as community halls, followed up with confirmed cases to ensure these individuals attended appointments at health facilities, and rechecked their BP status periodically. Another group of volunteers was recruited to provide BP screening at non-traditional but convenient locations such as coffee shops, dentists’ offices, marketplaces, neighbourhood security posts, and tailor shops. These volunteers were termed ‘BP checkpoints’, and they each received a digital BP monitor as well as training on hypertension knowledge and communication, how to correctly measure BP (based on WHO guidelines on how to take and record BP measurement), and how to set up and run a BP checkpoint in the community. After the volunteers’ training, CH2 supported the continuation of their consistent, high-quality hypertension screening and referral services through ongoing supportive supervision, job aids, and guidelines. Monthly monitoring and data quality assurance processes by CHS staff showed that collaborators and BP checkpoints continued to correctly measure BP^18,22^.

Like the collaborators, the BP checkpoints referred people with high BP readings to the health system for follow-up. CHS staff were also responsible for managing, guiding, and monitoring collaborators and BP checkpoints to reach out to individuals with elevated BP readings who had not presented at a health care facility for further follow up.

To further extend the reach of PHC services and strengthen the sustainability of the programme, CH2 partnered with pharmacies and social enterprises (SEs). CH2 trained the SEs on technical and monitoring and evaluation skills, sales and online marketing skills, and delivery of hypertension services. CH2 supported the SEs to develop financially sustainable business plans to provide community-based screening linked to diagnosis, treatment, and follow-up. The SEs worked with local markets, public parks, and small businesses to establish BP checkpoints and sell electronic BP cuffs for a commission. They set up a hypertension counselling hotline. CH2 also launched a partnership with the largest pharmacy chain in Vietnam—Pharmacity—which has 180 stores (mostly in Ho Chi Minh City) and aims to have 500 nationwide by 2023. CH2 worked with the pharmacy’s training departments to develop programmes and provided them with social and behaviour change communication materials. As a result, 121 staff were trained to provide in-store BP measurements, provide health promotion and disease prevention messages, and refer patients diagnosed with hypertension to health facilities. Services were promoted online, in the stores, and in Pharmacity’s monthly magazine.

To support these new patients, CH2 strengthened the capacity of public and private hospitals, CHS providers, and community networks based on Ministry of Health and WHO guidelines. This included developing and training a network of health facilities to provide standardised, high-quality services; creating standardised training packages; and fostering clinical quality by developing a team of trainers and quality assurance leaders that included doctors, nurses, and other NCD staff. These capacity-building efforts encompassed a network of 79 health facilities that provided standardised services (Table 1).

**Table 1:** Facilities where staff were trained by Communities for Healthy Hearts to provide standardised hypertension services.

District	Public hospitals	District health centres	Private clinics	Commune health stations	Total
District 8	1	1	2	16	20
District 12	1	1	4	11	17
Go Vap District	1	1	7	16	25
Thu Duc District	2	1	2	12	17
Total	5	4	15	55	79

Treatment adherence and self-care was supported through targeted face-to-face interventions by community volunteers, SE staff, and health care workers, along with a suite of BP self-care tools such as free BP checks, a BP diary, and short message service (SMS) texts, including appointment reminders and healthy lifestyle guidance.

**Figure 3:**
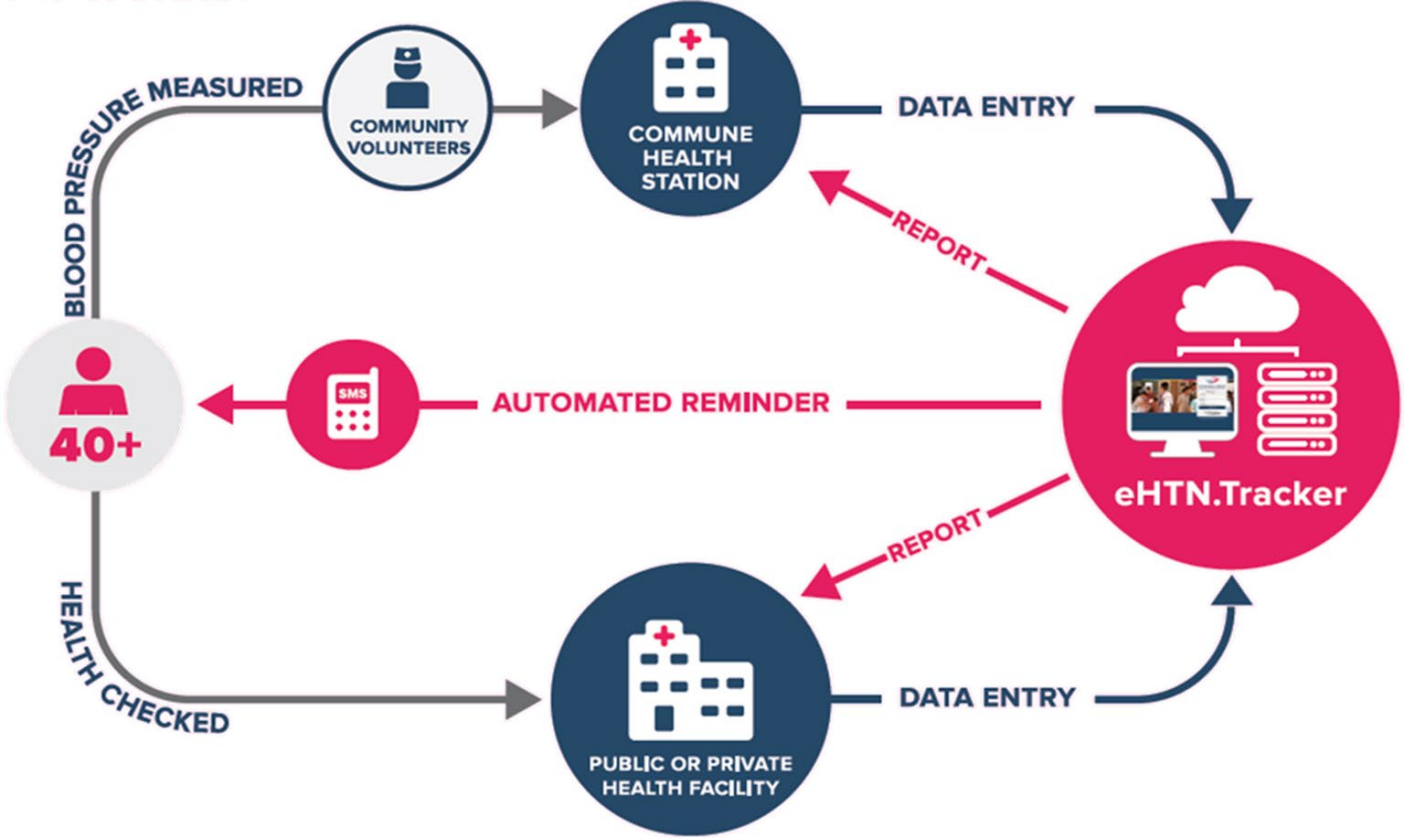
Flow chart of Communities for Healthy Hearts hypertension digital patient registry, the eHTN.Tracker. *Source*: PATH.

To facilitate follow-up of clients detected with elevated BP, CH2 developed a hypertension digital patient registry, the eHypertension.Tracker (eHTN.Tracker)^23^, a searchable online database containing data on hypertension screening, diagnosis, care, and treatment, for use at CHSs. This tool made it possible for CHSs to generate monthly electronic reports on the hypertension screening, diagnosis, and treatment cascade; to keep lists of people referred for diagnosis; and to record requirements for ongoing care and support. The eHTN.Tracker supported collaborators, BP checkpoints, and health workers at all levels to provide individualised support on a case-by-case basis and to more easily collate data to gain a fuller picture of the hypertension burden in their areas. The eHTN.Tracker also helped generate weekly SMS texts for hypertension patients to promote retention in care, treatment adherence, and healthy lifestyles. Figure 3 shows the support provided by this tool.

The project trained 55 health workers at CHSs (1 per CHS) to use the eHTN.Tracker. These health workers logged all clients into the eHTN.Tracker with an initial BP measurement and continued to follow them over time. Demographic characteristics of the 30,446 clients with elevated BP at screening or previous diagnosis of hypertension entered in the eHTN.Tracker are shown in Table 2.

**Table 2:** Client awareness of hypertension status by district at baseline and endline.

	District 8 (n = 319) (%)	District 12 (n = 331) (%)	Go Vap (n = 322) (%)	Thu Duc (n = 324) (%)	Average across districts (n = 1296) (%)	P value
Baseline (January 2016)	47.5	45.0	49.3	46.7	47.1	0.001
Endline (May 2019)	57.5	70.4	64.8	62.0	63.7	0.001

To support awareness and risk reduction, CH2 implemented innovative behaviour change communication campaigns and leveraged multiple communication platforms such as television, social media, web platforms, and direct communication via collaborators, health workers, and family members to increase awareness of CVD prevention. Key messages about cardiovascular health included ‘Know your blood pressure’; ‘Regular blood pressure check—small act, high impact’; and ‘Reduce salt intake for hypertension prevention and control’.

### Programme evaluation

The CH2 programme evaluation measured the effectiveness of the project. The main outcome indicators were:

- Increased exposure to CVD prevention messages.
- Increased availability of hypertension screening services.
- Increased hypertension awareness.
- Increased retention in care.
- Improved BP control for those on treatment.

The evaluation team used data from project reports and the eHTN.Tracker, including the number of people screened, diagnosed, treated, and achieving BP control. Relevant data from December 2016 through July 2019 were accessed in November 2019. All data were anonymously aggregated to protect privacy and confidentiality.

For assessment of client awareness of hypertension status, the required sample size was calculated for a before-and-after study based on the McNemar’s test, with p-before set at 50% and p-after set at 75%^22^. For the baseline survey, the same number of adults of each sex and age group in each district were included, for a total of 1296 participants. The same individuals were recruited for the endline survey.

Descriptive statistics were used, with frequencies and percentages reported for categorical variables. To detect differences in the systolic and diastolic blood pressure means of hypertension clients before and after interventions, Paired Samples T-Test, Fisher’s exact tests, and Chi-square test were used when appropriate for categorical variables. A significance level of 0.05 was used for all statistical tests. All analyses were carried out using SPSS Statistics package v20.

## Results

### Increased exposure to CVD prevention messages

CH2 used multiple communication platforms—both electronic and in person—to increase awareness of CVD prevention. The programme estimates that messages designed to educate, motivate, and support people to seek hypertension screening and treatment and make healthy lifestyle changes had reached more than 1.28 million people through these platforms by the end of November 2019. Since NCDs were not previously prioritized and hence there was lack of public messaging and awareness campaigns targeting hypertension management, we assume that people were not receiving these messages prior to the start of the campaign.

### Increased availability of hypertension screening services

CH2 recruited 135 collaborators from the community across the project area to screen and follow up with clients in their homes or at community halls. In addition, the project recruited 357 volunteers at non-traditional checkpoints such as small businesses and 48 volunteers from SE partners. Thus, there were 540 new BP screening access points (an average of 35 screening access points per commune with an average of 15,000 people each), an increase of 34 times the baseline number of 16 screening points, previously only at the CHSs. (These 16 screening points were available during the project as well and were used by some collaborators).

From June 2016 until the end of the project in 2019, 121,273 (58%) of adults aged 40 years and older in the project catchment area had received an initial hypertension screening^17^. By comparison, before the project intervention, only 2350 people were screened in 12 months by CHSs. Amongst those screened during the project, 82.0% were screened by collaborators and BP checkpoints managed by CHSs, 12.5% by checkpoints managed by SE partners, and 5.5% by health workers at CHSs.

### Increased hypertension awareness

As a result of the increased screening and linkage to health facilities with access to trained personnel, awareness of hypertension status amongst participants increased significantly. Table 3 shows that before the project, 47.1% of people surveyed at baseline knew their hypertension status. After the project, this proportion increased to 63.7%.

**Table 3:** Profiles of clients registered in the e.HTN.Tracker.

Characteristics	n	%
**Total**	30,446	100.0
**Location**		
Thu Duc	5445	17.9
Go Vap	6177	20.3
District 8	8784	28.9
District 12	10,040	33.0
**Age group**		
40–49	6720	22.1
50–59	8845	29.1
60–70	8991	29.5
70+	5890	19.3
**Gender**		
Male	13,975	45.9
Female	16,471	54.1
**Have a phone number**		
Yes	24,579	80.7
No	5867	19.3
**Have a blood pressure monitor at home**		
Yes	3626	11.9
No	26,820	88.1
**Hypertension diagnosis known before contact**		
Yes	14,980	49.2
No	15,466	50.8

### Increased retention in care

**Figure 4:**
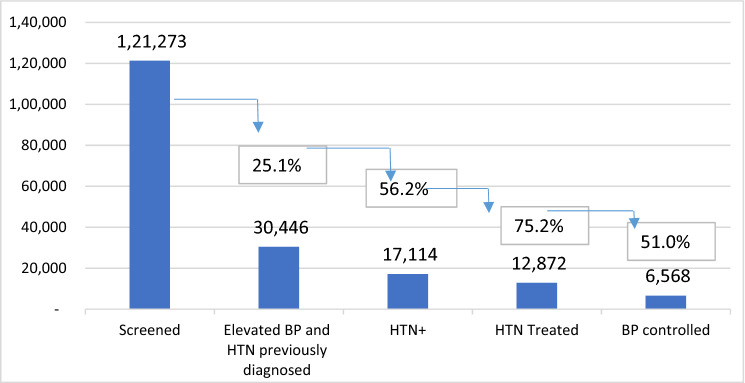
Proportion of Communities for Healthy Hearts clients screened, diagnosed, and treated for hypertension. *Source*: eTracker, PATH, 2016–2019.

Retaining clients in care was supported by SMS messages, digital tracking, and targeted case management by community volunteers. Of the 121,273 adults screened, 30,446 (25.1%) showed elevated BP or were identified as previously diagnosed with hypertension and were entered into the eHTN.Tracker. All were referred for diagnosis or re-engagement with treatment. Of this group, 17,114 (56.2%) were subsequently diagnosed with hypertension, and 12,872 (75.2%) of these were placed on treatment. By the end of the project, 6,568 (51.0%) of those on treatment were successfully controlling their BP. Considered more broadly, of the 17,114 people initially confirmed with hypertension, 6,568 (38.4%) had their BP controlled at endline (Fig. 4).

### Improved blood pressure control for those on treatment

Of the 12,872 people treated for high BP, the study followed 11,877 (92.3%) to endline. Of those, the proportion experiencing high BP was significantly reduced: 73.2% versus 58.3% (Table 4). In addition, their mean systolic and mean diastolic BP readings had decreased.

**Table 4:** Change in blood pressure from baseline to last visit (n = 11,877).

	Baseline	Endline	Change	*p* value
Proportion with high blood pressure (SP ≥ 140 and/or DP ≥ 90)	73.2%	58.3%	− 14.9%	*p* < 0.005
Mean SP	142 mmHg	140 mmHg	− 2	*p* < 0.0001
Mean DP	88 mmHg	87 mmHg	− 1	*p* < 0.0001

*DP* diastolic pressure, *SP* systolic pressure.

## Discussion

Many people in Vietnam and other low- and middle-income countries are unaware of their hypertension status. As a result, diagnosis often comes late in the disease process, when the condition is acute and harder to manage. In CH2 intervention areas, early diagnosis, greater proportion of patients initiating treatment, and improved BP control were supported by system-level interventions. The CH2 programme brought together solutions that have shown promise in other countries, yet our model was unique in its application to the hypertension continuum of care, its implementation in Vietnam, and its combination of and interplay between interventions. The combined interventions that made up the CH2 model were a first step in Vietnam towards a differentiated care approach for NCDs that will be sustainable and scalable.

The programme further enhanced adherence to treatment regimens via tools such as SMS and BP diaries^24–26^. This comprehensive and evidenced-based approach reflected recommendations from WHO guidelines and frameworks^2^. The eHTN.Tracker in particular provided data to focus and tailor efforts of community-based services on those who required the greatest support to adhere to treatment, including attending follow-up appointments. Prepared health workers further supported the provision of standardised treatment and retention in care.

The role of community volunteers in strengthening linkage to care and adherence to treatment is well documented in low- and middle-income countries across infectious and non-infectious diseases^25–29^—yet before CH2, screening for hypertension in Vietnam was primarily provided as part of a costly physical exam. By engaging local volunteers and private-sector partners, CH2 made screening and follow-up quick and convenient and reduced associated costs, such as transportation and time off work. This approach vastly increased the percentage of people in the target age group reached compared with hospital or facility-based screening: 58% versus 10%, as reported in the 2015 STEPS survey^12^.

Similarly, CH2 study results indicated that a greater percentage of those diagnosed with hypertension initiated treatment and achieved BP control than was found in previous studies. In the CH2 project, the percentage of people with hypertension who initiated treatment was 75.2%, whereas only 24.9% of those diagnosed were treated according to the STEPS survey analysis^12^ and 32.5% in a similar study in Ghana in 2019^30^. Another national study in Vietnam showed that 29.6% of those diagnosed received treatment^11^, and a study in central Vietnam showed that 33.2% of participants were treated^8^. Although these studies showed that 10.7% and 12.2%, respectively, of those with hypertension had their BP controlled, CH2 results showed that 38.4% of patients with hypertension were able to control their BP, with statistically significant reductions in systolic and diastolic measures at levels associated with reduced CVD risk. Comparing the percentage of patients with their BP controlled amongst those treated, the CH2 project results were higher than in the two other surveys: 51.0% versus 36.3% and 36.8%, respectively^8,11^. However, it should be noted that despite the project’s success in reducing those experiencing high BP (from 73.2% to 58.3% among those treated for high BP and followed to endline), it still indicates a large number of individuals at risk. Further study should be conducted to better understand the reasons for this and strategies for risk reduction.

In a recent quasi-experimental study, CH2 interventions were found to significantly increase BP self-management activities such as accessing free BP checks, using BP diaries, and receiving and responding to SMS messages. Retention rates and adherence to treatment exceeded what was found in other studies, and the evidence suggests that the focus on self-care and treatment adherence contributed to these results as well as enabling people living with hypertension to manage their BP^31^.

The COVID-19 pandemic has further highlighted the need for an inclusive strategy to build resilient health systems. Interventions such as those tested in CH2 provide valuable lessons learned to shape the way forward.

### Limitations of the evaluation

Our findings provided evidence for the effectiveness of the model intervention in reaching adults from lower-income households in several districts of Ho Chi Minh City, yet this approach and results may not be generalisable to areas with different demographics. We used an opportunistic sample of those who sought the services of the CH2 programme and were retained in care to the end of the project period, which may have resulted in greater representation of individuals motivated to improve their health. The mechanisms through which positive outcomes were achieved were not determined as part of this study. Evaluation of the eHTN.Tracker data was in effect a large cohort study, with a limitation that it did not have a control group regarding the findings on BP control.

## Conclusion

Although committed to improving hypertension management, Vietnam has struggled with gaps in the hypertension care cascade. The CH2 programme, in keeping with WHO 2015 STEPS survey recommendations, introduced an innovative service delivery model to address these gaps, based on local needs and international best practices. Our results indicated solid gains in early diagnosis, increase in treatment, and improved BP control amongst those treated.

Building on the success of the programme, PATH, and the Vietnam Ministry of Health, in partnership with Access Accelerated, are scaling these interventions nationally, focusing on hypertension and diabetes and retaining the community-based programming and linkage to health facilities. Lessons learned from the digital solutions used in this project are being integrated into a national health information system for NCDs, and two apps—one for people living with NCDs and one for health workers—are being added to the suite of supportive tools. In addition, the Ho Chi Minh City Municipal Health Department, which is committed to extending the community-based approach and realising the potential of public–private partnerships as demonstrated in CH2, recently launched a collaboration with Saigon Co.op to raise awareness of CVD risk and offer hypertension screening services in supermarkets across the province. These local activities and innovations show a continued commitment to scale and sustain the interventions introduced through CH2.

## Data Availability

The datasets generated during and/or analysed during the current study are available from the corresponding author on reasonable request.

## References

[1] World Health Organization. *Cardiovascular Diseases (CVDs)*. http://www.who.int/mediacentre/factsheets/fs317/en/ (2017).

[2] World Health Organization. *HEARTS Technical Package*. https://www.who.int/publications/i/item/hearts-technical-package (2018).

[3] World Health Organization (WHO). *Tackling NCDs: ‘Best Buys’ and Other Recommended Interventions for the Prevention and Control of Noncommunicable Diseases* (WHO, 2017). http://apps.who.int/iris/bitstream/10665/259232/1/WHO-NMH-NVI-17.9-eng.pdf?ua=1.

[4] World Economic Forum, World Health Organization. *From Burden to ‘Best Buys’: Reducing the Economic Impact of Non-communicable Diseases in Low- and Middle-Income Countries* (Working paper series) (World Economic Forum, 2011). https://www.who.int/nmh/publications/best_buys_summary.pdf.

[5] World Health Organization. *Hypertension*. https://www.who.int/news-room/fact-sheets/detail/hypertension (2019).

[6] World Health Organization (WHO) (2018). Noncommunicable Diseases Country Profiles 2018.

[7] Meiqari L, Essink D, Wright P, Scheele F (2019). Prevalence of hypertension in Vietnam: A systematic review and meta-analysis. Asia Pac. J. Public Health..

[8] Hien HA, Tam NM, Tam V, Derese A, Devroey D (2018). Prevalence, awareness, treatment, and control of hypertension and its risk factors in (Central) Vietnam. Int. J. Hypertens..

[9] Pham TX (2017). Reality of hypertension in aged of 45 - 64 year in Dien Bien district in 2014. J Prev Med..

[10] Hoang VM (2019). Patterns of raised blood pressure in Vietnam: Findings from the WHO STEPS Survey 2015. Int. J. Hypertens..

[11] Son PT (2012). Prevalence, awareness, treatment and control of hypertension in Vietnam—Results from a national survey. J. Hum. Hypertens..

[12] Vietnam Ministry of Health, General Department of Preventive Medicine (2016). National Survey on the Risk Factors of Non-communicable Diseases (STEPS) Viet Nam, 2015.

[13] Vietnam Ministry of Health (2015). National Strategy on Prevention and Control of NCDs, 2015–2025.

[14] Vietnam Ministry of Health (2016). Plan for People’s Health Protection, Care and Promotion 2016–2020.

[15] Cheng T-M (2014). Vietnam’s health care system emphasizes prevention and pursues universal coverage. Health Aff..

[16] Poudel KC, Fujita M, Green K, Poudel-Tandukar K, Jimba M (2011). Non-communicable diseases in southeast Asia. Lancet.

[17] Central Population and Housing Census Steering Committee (2020). Results: The Viet Nam Population and Housing Census of 00:00 hours on 1 April 2019.

[18] Tran TA (2020). Strengthening local health systems for hypertension prevention and control: The Communities for Healthy Hearts program in Ho Chi Minh City, Vietnam. J. Glob. Health Sci..

[19] Tran, H. *et al.**Communities for Healthy Hearts: Participant Assessment*. (2016).

[20] Barr VJ (2003). The expanded Chronic Care Model: An integration of concepts and strategies from population health promotion and the Chronic Care Model. Hosp. Q..

[21] World Health Assembly (2016). Framework on Integrated, People-Centred Health Services: Report by the Secretariat.

[22] Nguyen TNP (2020). Knowledge change related to hypertension in the Southern province of Vietnam: A community based, before and after intervention evaluation. J. Glob. Health Sci..

[23] Communities for Healthy Hearts. *eHTN.Tracker*. www.ch2.vn (2018).

[24] Joshi R, Jan S, Wu Y, MacMahon S (2008). Global inequalities in access to cardiovascular health care: Our greatest challenge. J. Am. Coll. Cardiol..

[25] Joshi R (2014). Task shifting for non-communicable disease management in low and middle income countries—A systematic review. PLoS ONE.

[26] Newman PM (2018). Community health workers improve disease control and medication adherence among patients with diabetes and/or hypertension in Chiapas, Mexico: An observational stepped-wedge study. BMJ Glob. Health.

[27] Sankaran S (2017). An NGO-implemented community–clinic health worker approach to providing long-term care for hypertension in a remote region of southern India. Glob. Health Sci. Pract..

[28] Wadler BM, Judge CM, Prout M, Allen JD, Geller AC (2011). Improving breast cancer control via the use of community health workers in South Africa: A critical review. J. Oncol..

[29] Ong’ang’o JR, Mwachari C, Kipruto H, Karanja S (2014). The effects on tuberculosis treatment adherence from utilising community health workers: A comparison of selected rural and urban settings in Kenya. PLoS ONE.

[30] Adler AJ (2019). Can a nurse-led community-based model of hypertension care improve hypertension control in Ghana? Results from the ComHIP cohort study. BMJ Open.

[31] Khuong QL (2020). Effect of community-based intervention on self-management of blood pressure among hypertensive adults: Findings from the Communities for Healthy Hearts quasi-experimental study in Vietnam. J. Glob. Health Sci..

